# COVID-19 inactivated and non-replicating viral vector vaccines induce regulatory training phenotype in human monocytes under epigenetic control

**DOI:** 10.3389/fcimb.2023.1200789

**Published:** 2023-07-14

**Authors:** Mateus da Silva Matias Antunes, Fabricia Heloisa Cavicchioli Sugiyama, Humberto Doriguetto Gravina, Ricardo Cardoso Castro, Francisco Javier Romero Mercado, Julia Oliveira de Lima, Caroline Fontanari, Fabiani Gai Frantz

**Affiliations:** Laboratory of Immunology and Epigenetics, School of Pharmaceutical Sciences of Ribeirão Preto, University of São Paulo, Ribeirão Preto, Brazil

**Keywords:** vaccine, COVID-19, trained immunity, monocyte, epigenetic

## Abstract

**Background:**

Trained immunity is the enhanced innate immune response resulting from exposure to pathogens or vaccines against an unrelated pathogen stimulus. Certain vaccines induce a memory like response in monocytes and NK cells, leading to modulation in cytokine production, metabolic changes, and modifications in histone patterns. Here, we hypothesized that vaccination against SARS-CoV-2 could induce the training of monocytes in addition to stimulating the adaptive immune response.

**Methods:**

Therefore, we aimed to investigate the immunophenotyping, cytokine and metabolic profile of monocytes from individuals who were completely immunized with two doses of inactivated COVID-19 vaccine or non-replicating viral vector vaccine. Subsequently, we investigated the epigenetic mechanisms underlying monocyte immune training. As a model of inflammatorychallenge, to understand if the monocytes were trained by vaccination and how they were trained, cells were stimulated *in vitro* with the endotoxin LPS, an unrelated stimulus that would provoke the effects of training.

**Results:**

When challenged *in vitro,* monocytes from vaccinated individuals produced less TNF-α and those who received inactivated vaccine produced less IL-6, whereas vaccination with non-replicating viral vector vaccine induced more IL-10. Inactivated vaccine increased classical monocyte frequency, and both groups showed higher CD163 expression, a hallmark of trained immunity. We observed increased expression of genes involved in glycolysis and reduced IRG1 expression in vaccinated subjects, a gene associated with the tolerance phenotype in monocytes. We observed that both vaccines reduced the chromatin accessibility of genes associated with the inflammatory response, the inactivated COVID-19 vaccine trained monocytes to a regulatory phenotype mediated by histone modifications in the *IL6* and *IL10* genes, while the non-replicating viral vector COVID-19 vaccine trained monocytes to a regulatory phenotype, mediated by histone modifications in the *IL6, IL10, TNF*, and *CCL2* genes.

**Conclusions:**

Our findings support the recognized importance of adopting vaccination against SARS CoV-2, which has been shown to be effective in enhancing the adaptive immune response against the virus and reducing mortality and morbidity rates. Here, we provide evidence that vaccination also modulates the innate immune response by controlling the detrimental inflammatory response to unrelated pathogen stimulation.

## Introduction

The pathogenic epitopes in vaccines stimulate adaptive immune cells, resulting in cellular and humoral immune responses that can be sustained for years by maintaining memory cells in the body (Nicholson, 2016). In addition to inducing adaptive immune memory, another mechanism, called trained immunity, is triggered by vaccines such as Bacillus Calmette–Guérin (BCG) and the yellow fever vaccine (Netea et al., 2011; Saeed et al., 2014; Bekkering et al., 2016). This mechanism utilizes the ability of monocytes and natural killer (NK) cells to respond better to a second, non-specific heterologous stimulus. It has been associated with epigenetic modifications in regions that promote the immune response, immunometabolism, and remodeling of cellular energy metabolism towards aerobic glycolysis (Cheng et al., 2014), which may increase oxidative phosphorylation (Arts et al., 2016; Netea et al., 2016). Innate immune cells can be trained by ligands of NOD2 or dectin-1 receptors (van der Meer et al., 2015), which may result in a trained cell with a pro-inflammatory (Quintin et al., 2012; Kleinnijenhuis et al., 2014b) or regulatory (Quinn et al., 2019; Debisarun et al., 2021) phenotype, depending on the stimulus.

Pro-inflammatory-trained cells are characterized by increased production of pro-inflammatory cytokines such as interleukin (IL)-6, IL-18, tumor necrosis factor alpha (TNF-α) (Kleinnijenhuis et al., 2012; Quintin et al., 2012; Rizzetto et al., 2016), and, in some cases, IL-1β, and an improved ability to kill pathogens such as *Candida albicans*, *Staphylococcus aureus*, and *Escherichia coli* (Kleinnijenhuis et al., 2014a; Rizzetto et al., 2016; Arts et al., 2018). In addition, pro-inflammatory trained cells show increased expression of SET7 protein, which causes an increase in the expression of the enzymes MDH2 and SDHB, both of which are involved in producing cellular energy in the Krebs cycle, promoting the accumulation of metabolites that promote oxidative phosphorylation and, consequently, the production of pro-inflammatory cytokines (Keating et al., 2020). Alternatively, regulatory trained cells are characterized by long-lasting enhanced anti-inflammatory responsiveness (Cauchi and Locht, 2018), such as increased production of IL-10 and IL-1Ra, both immune regulatory cytokines (Quinn et al., 2019; Guha et al., 2021), and reduced production of IL-6, TNF-α, and IL-1β (Guha et al., 2021; Wimmers et al., 2021). In addition, human monocytes trained in the regulatory profile expressed more CD163 and CD206, which promoted inflammation resolution (Guha et al., 2021).

Considering the ability of vaccines to provoke the training phenotype of monocytes, which initiate an immune response to a vaccine antigen via cytokine and chemokine production and antigen presentation, the underlying mechanisms are important to improve vaccine technology and evaluate the potential benefits of immunization. In this context, several vaccines for coronavirus disease 2019 (COVID-19) were developed due to recent health emergency, and the protective role of the innate immune system in vaccination remains to be explored. Here, we showed that vaccination against COVID-19 with a non-replicating viral vector vaccine (nRVVac) and with an inactivated vaccine (InVac) may trigger a training phenotype during the first months following immunization. Both vaccines evaluated in this study induced innate training, and we suggest that the training was characterized by a regulatory phenotype that could be crucial for controlling the exacerbated inflammatory response in further COVID-19 infection. Our results support the widespread vaccination and reinforce its value, particularly considering its effectiveness in modulating epigenetically the innate immune response.

## Results

### Non-replicating viral vector vaccine increases and sustains CD163 expression via classical and intermediate monocytes

To determine whether nRVVac and InVac modulate the monocyte phenotype, we evaluated the expression of a set of surface markers before and after lipopolysaccharide (LPS) *in vitro* challenge. The trained cell is characterized by the ability of the cell to respond sharply to a heterologous stimulus, not correlated to the first one. Usually, in training protocols, LPS is used as a challenge to evaluate the cell response, due to the extensive knowledge about its mechanism of action and the expected response (Netea et al., 2020).

**Figure 1:**
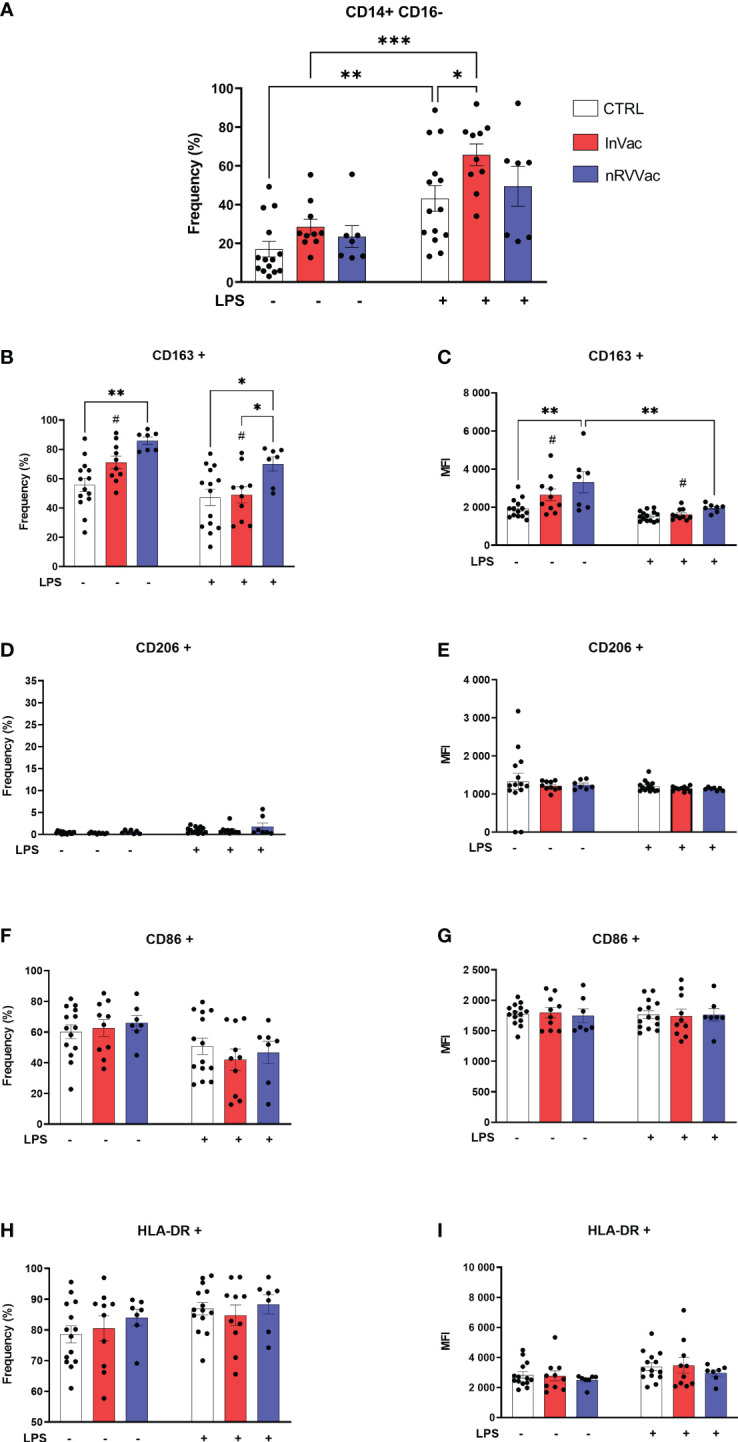
Vaccination against COVID-19 modulates the frequency of classical monocytes and the expression of their membrane markers. Monocytes isolated from peripheral blood of CTRL, InVac, and nRVVac groups and challenged *in vitro* with LPS to assess the training profile. **(A)** Representative dotplot of distribution and frequency of CD14+CD16− classical monocytes. **(B)** Percentage of CD14+CD16− expressing CD163 and **(C)** mean fluorescent intensity (MFI) of CD14+CD16− expressing CD163; **(D, E)** CD206; **(F, G)** CD86; and **(H, I)** HLA-DR. Data were expressed as means ± SEM of 14, 10, and 7 individual CTRL, InVac, and nRVVac donors, respectively. Statistical analysis was performed using two-way ANOVA with Tukey’s post-test. **^#^***p ≤* 0.05 between InVac and InVac+LPS, *p ≤ 0.05, **p ≤ 0.01, ***p ≤ 0,001. Unvaccinated controls (CTRL), vaccinated with inactivated vaccine (InVac), and non-replicating viral vector vaccine (nRVVac).

Classical monocytes are generally committed to phagocytosis, adhesion, and migration and are characterized by the expression of CD14++CD16−. Flow cytometry revealed that the frequency of classical monocytes increased after *in vitro* stimulation with LPS in the control (CTRL) and InVac groups, and this increase was greater in the InVac group than in CTRL (**Figure 1A**). Furthermore, when co-stimulatory and co-receptor molecules were evaluated, the average frequency of CD86 and HLA-DR expression was above 60%; however, no differences were observed between the groups (**Figures 1F–I**), while CD206 expression was not detected (**Figures 1D, E**). Additionally, we observed an increase in the fraction of CD163 in classical monocytes from the nRVVac group when the cells were stimulated with LPS, whereas only the InVac group showed a reduction in CD163 expression after LPS stimulation (**Figure 1B**). Furthermore, only the nRVVac group showed an increase in the mean fluorescence intensity (MFI) of CD163 in the non-stimulated group; however, when challenged with LPS, both vaccinated groups showed reduced MFI of this marker to CTRL levels (**Figure 1C**).

**Figure 2:**
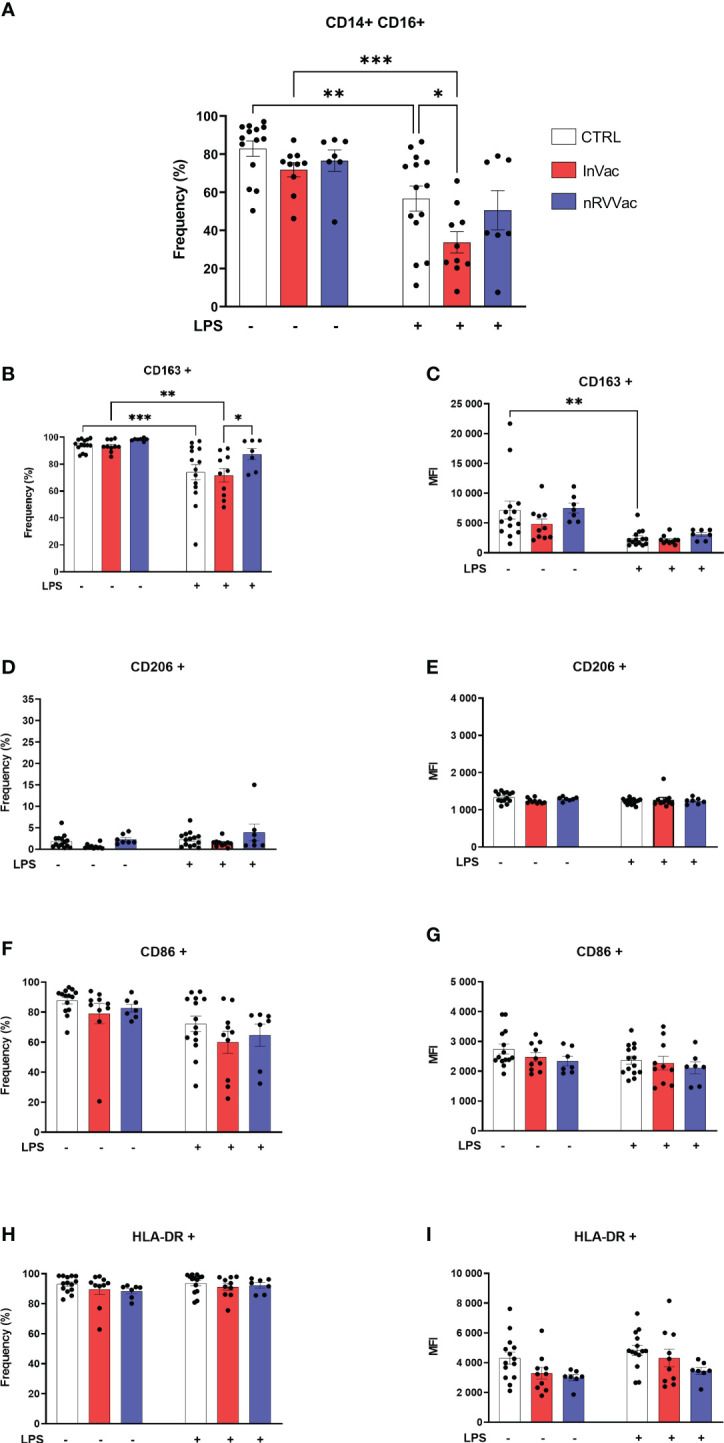
Inactivated vaccine against COVID-19 modulates the frequency of intermediate monocytes and the expression of their membrane markers. Monocytes were isolated from peripheral blood of CTRL, InVac, and nRVVac groups and challenged *in vitro* with LPS to assess the training profile. **(A)** Representative dotplot of distribution and frequency of CD14+CD16− classical monocytes. **(B)** Percentage of CD14+CD16+ expressing CD163 and **(C)** MFI of CD14+CD16+ expressing CD163; **(D, E)** CD206; **(F, G)** CD86; **(H, I)** HLA-DR. Data were expressed as means ± SEM of 14, 10, and 7 individual CTRL, InVac, and nRVVac donors, respectively. Statistical analysis was performed using two-way ANOVA with Tukey’s post-test. * *p ≤* 0.05, **p ≤ 0.01, ***p ≤ 0,001. Data are shown as means ± SEM.

Intermediate monocyte levels, characterized by the expression of CD14 and CD16 (CD14+ CD16+), were reduced after stimulation with LPS in the CTRL and InVac groups. This reduction was more pronounced in the InVac group than in the CTRL group (**Figure 2A**). Similarly, in classical monocytes, HLA-DR frequency levels remained unaltered and were close to 90% of all cells, and CD206 was not expressed regardless of the group or stimulus (**Figures 2D, E, H, I**). In addition, intermediate monocytes from all groups expressed high levels of CD163, which were reduced after stimulation with LPS in the CTRL and InVac groups but remained constant in the nRVVac group (**Figures 2B, C**). Only the vaccinated groups sustained CD163 levels after stimulation with LPS; its expression was reduced in the CTRL group. Considering that intermediate monocytes accounted for almost 60% of the total monocytes in the CTRL group, the loss of CD163 in this population demonstrates a drastic reduction in this marker in non-vaccinated individuals. The expression of CD86 were not modulated in intermediate monocytes (**Figures 2F, G**).

**Figure 3:**
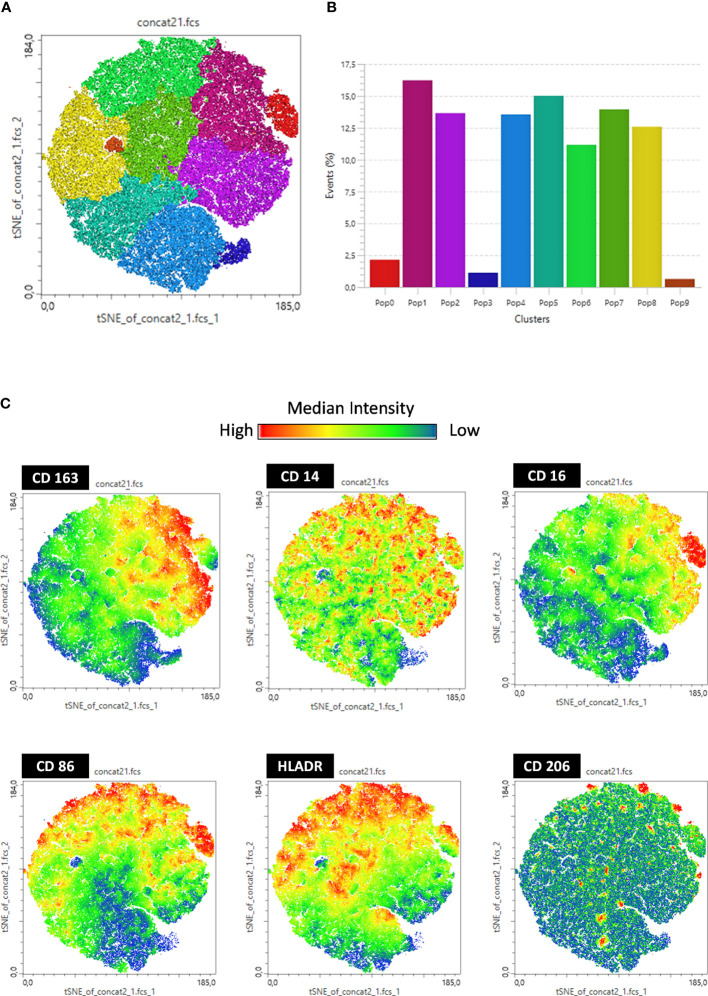
t-SNE-guided analysis of monocytes. All cytometry data were merged to create a single t-SNE map with the signal strength of six phenotypic markers. **(A)** Cell populations defined by the manual gating strategy shown in **Figures 1**, **2** are projected onto t-SNE software and assigned specific colors based on frequency of population. **(B)** Frequency projections of identified clusters are represented by bars. **(C)** Frequency projection of identified clusters are represented by geographical distribution in heat scale. Each individual marker is represented with a blue–green–red continuous color scale, wherein the warmer the color, the greater the expression of the surface marker.

**Figure 4:**
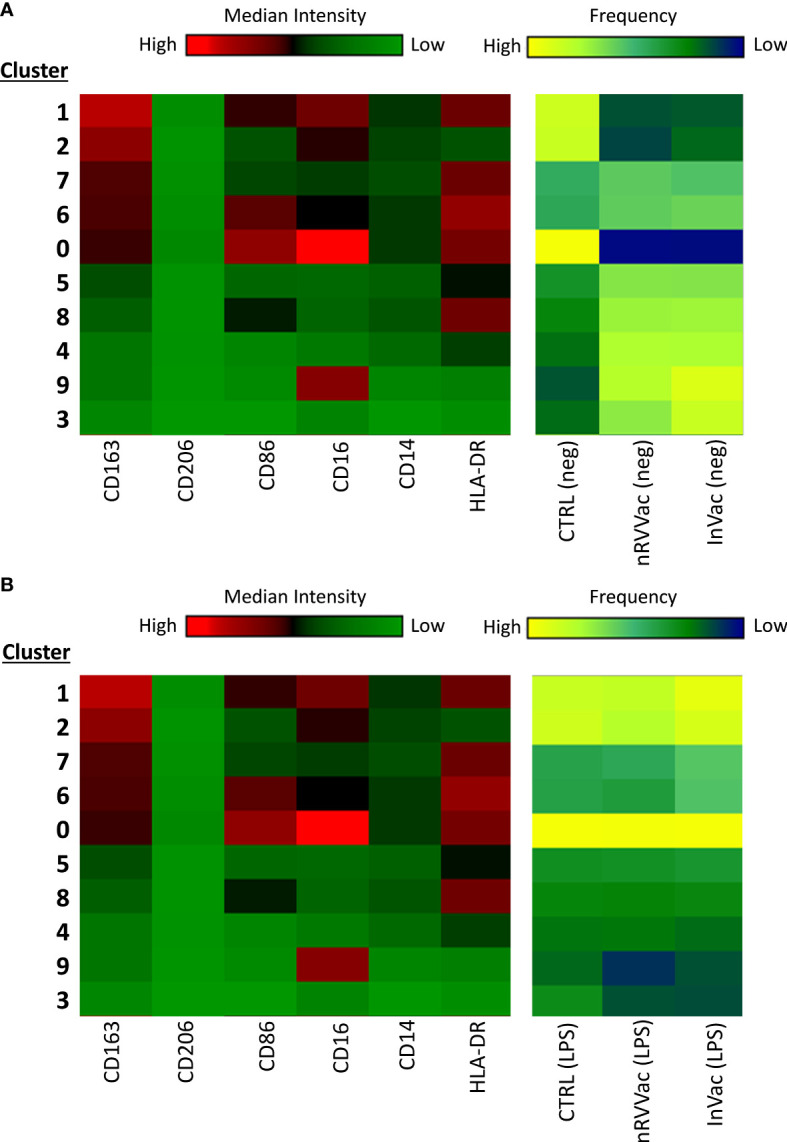
Heatmap graph indicating the expression of surface markers according to the different clusters identified using tSNE analysis, before and after LPS stimulation. At the left end are arranged all 10 clusters identified using tSNE analysis, ordered according to CD163 expression, in descending order. At the right end, the three columns represent the CTRL, nRVVac, and InVac groups. The expression level of the markers is represented with the green–black–red continuous color scale, where the warmer the color, the greater the expression. Similarly, the frequency of each cluster is represented by a blue–green–yellow continuous color scale, where the warmer the color, the greater the frequency of the analyzed cluster. **(A)** Heatmap of tSNE analysis before stimulation with LPS (neg). **(B)** Heatmap of tSNE analysis after stimulation with LPS (LPS).

In addition, we performed t-distributed stochastic neighbor embedding (tSNE) analysis based on the frequency and density of expression of different cell markers. We found 10 monocyte subpopulations (Pop) numbered from 0 to 9 (**Figures 3A, B**). These subpopulations are composed of all events acquired on flow cytometry expressing the CD14+ monocyte marker. Therefore, it is a mixture of classical and intermediate monocytes that can be discriminated through CD16 expression in **Figure 4**. The tSNE global projection of monocyte surface markers is represented in the geographical map on a heat scale (**Figure 3C**). Clusters 1 and 5 had the highest percentages of events. Furthermore, clusters 2, 4, 6, 7, and 8 constituted 11%–15% of the analyzed events. Finally, clusters 0, 3, and 9 were the least frequent (**Figure 3B**).

We then transformed the tSNE maps into heatmap graphics to visualize the clusters and differentiate between experimental groups, both non-stimulated and stimulated with LPS. Therefore, we organized the clusters according to CD163 expression in a descending order, separating the unstimulated (**Figure 4A**) and LPS-stimulated groups (**Figure 4B**).

Upon analyzing the heat maps, we observed that, before LPS stimulation, clusters 1 and 2 were more abundant in the CTRL group than in the vaccinated group, and both clusters expressed high levels of CD163. Cluster 1 also expressed high levels of CD16 and HLA-DR and moderate levels of CD86, typical of monocytes with antigen-presenting function and co-stimulation. Cluster 2 cells expressed moderate levels of CD16 and low levels of HLA-DR, which are typical of monocytes with patrolling and inflammation resolution functions. Both clusters showed higher numbers of vaccinated subjects following LPS stimulation.

Furthermore, cluster 0 is composed of monocytes that express high levels of CD86 and HLA-DR and are CD16 high, which is characteristic of intermediate monocytes, and is present only in the CTRL group before stimulation but increases in the vaccinated groups and is sustained at the same level as CTRL after stimulation.

In addition, clusters 7 and 6 expressed high levels of CD163 and HLA-DR and were less present in the CTRL group but were increased in the vaccinated groups. These clusters differed only in the expression of CD86 and CD16, which were both more highly expressed in subpopulation 6, making them closer to intermediate monocytes, and considerably reduced in subpopulation 7, resembling classic monocytes. Interestingly, after stimulation with LPS, these clusters appeared to be the same between groups, being slightly increased in the InVac group. Cluster 7, which refers to classical monocytes, had one of the highest frequencies among the identified clusters (**Figure 3B**), which agrees with the increase in the frequency of classical monocytes observed in InVac (**Figure 2A**).

**Figure 5:**
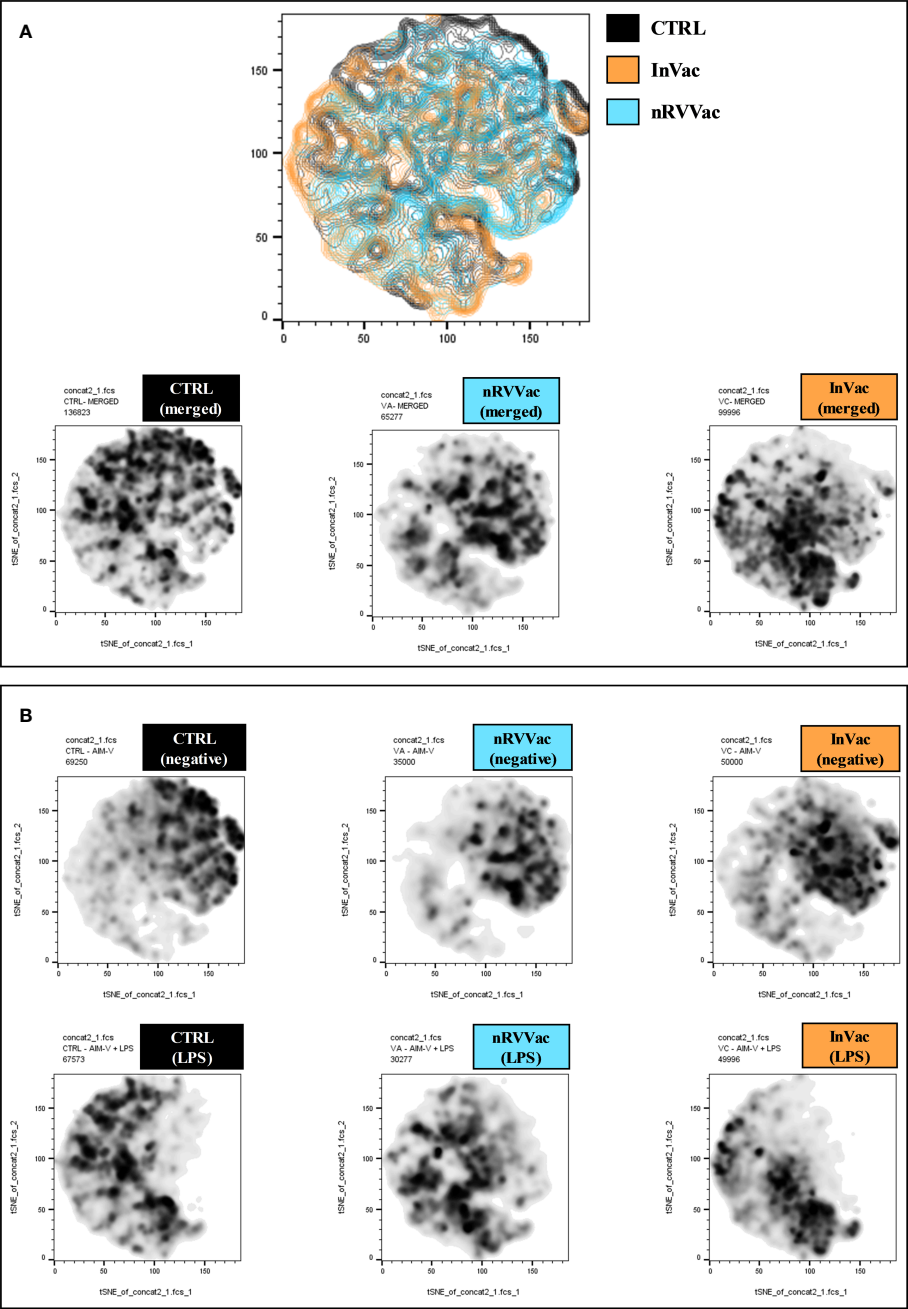
Geographical distribution of experimental groups on the tSNE plot. **(A)** The first graph shows the tSNE graph in contour plot and geographic distribution of CTRL (black), InVac (orange), and nRVVac (blue) groups. Graphs at the bottom represent areas occupied by monocytes from the CTRL, nRVVac, and InVac groups, respectively; both stimulated and non-stimulated (merged) are represented in density plots. **(B)** The graphs at the top of the figure represent the location of unstimulated monocytes (negative) on the tSNE graph, while the bottom graphs represent the location following LPS stimulation (LPS). After LPS stimulation, all groups presented a reduction in the number of cells in the regions that express high levels of CD163; however, this reduction is smaller in the nRVVac group.

Furthermore, before stimulation, clusters expressing low levels of CD163 (Pop 5, 8, 4, 9, and 3) were increased in vaccinated individuals compared to controls, but after stimulation, these clusters were considerably reduced in vaccinated individuals, and there was an increase in clusters 1, 2, and 0, which expressed high levels of CD163. By analyzing the regions on the tSNE graph populated by the CTRL, InVac, and nRVVac groups, we observed that monocytes from the nRVVac group were concentrated in areas that coincided with subpopulations 1, 2, 7, and 6 (**Figure 5A**). By analyzing the density plots that separated the monocytes before and after LPS stimulation, we observed the migration of the cell population to areas that expressed less CD163 after stimulation; however, migration was lower in the nRVVac group (**Figure 5B**). Based on these results, we suggest that classical and intermediate monocytes from the nRVVac group can sustain CD163 expression following LPS stimulation.

### Both vaccines induced a modulation on cytokines produced by challenged monocytes

**Figure 6:**
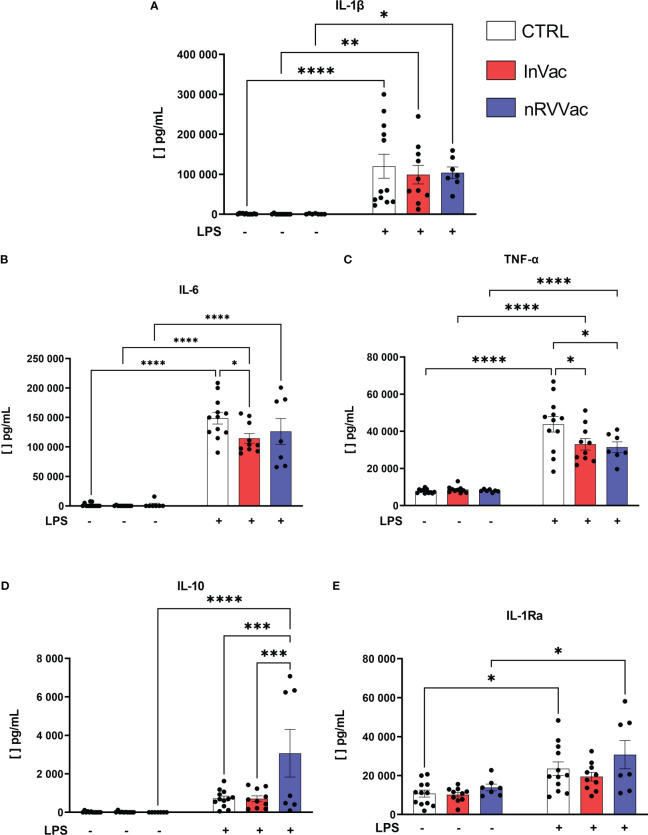
Cytokines released by monocytes before and after LPS stimulation. Monocytes were isolated from peripheral blood of CTRL, InVac, and nRVVac groups and challenged *in vitro* with 100 ng/ml of LPS for 6 h and the culture supernatant collected for quantifying cytokines using ELISA. **(A)** Representative dotplot of IL-1β, **(B)** IL-6, **(C)** TNF-α, **(D)** IL-10, and **(E)** IL-1Ra. Data were expressed as means ± SEM of 12, 10, and 7 individual CTRL, InVac, and nRVVac donors, respectively. The results were analyzed using one-way ANOVA with Šidák’s post-test. *p ≤ 0.05, **p ≤ 0.01, ***p ≤ 0.001, and ****p ≤ 0,0001.

To understand if both vaccines, besides changing the phenotypic profile of monocytes, are also modulating their capacity to produce cytokines, we evaluated the production of IL-1β, TNF-α, IL-6, IL-10, and IL-1Ra, all related to trained immunity. When stimulated with LPS, monocytes from the three groups showed increased IL-1β production; however, no differences were observed in IL-1β production between the groups (**Figure 6A**).

Furthermore, basal TNF-α production was constant in all non-stimulated groups, and *in vitro* stimulation with LPS increased TNF-α production. However, monocytes from vaccinated individuals showed reduced TNF-α production after stimulation with LPS, when compared to monocytes from non-vaccinated individuals (**Figure 6C**). Similarly, after stimulation with LPS, IL-6 production increased, and monocytes from individuals vaccinated with InVac showed reduced release of IL-6 (**Figure 6B**).

In addition, when stimulated with LPS, only the nRVVac group showed an increase in the average production of IL-10 (**Figure 6D**); the InVac group did not show an increase in IL-1Ra production in response to stimulation (**Figure 6E**).

### Non-replicating viral vector vaccine increases glycolysis and reduces IRG1 expression

Different metabolic pathways, including glycolysis, amino acid metabolism, and lipid metabolism, are modulated during trained immunity. Differences were observed in the basal production of HK2 and LDHA genes in nRVVac individuals (**Table 1**), indicating an increase in the glycolytic pathway. No changes were observed in the baseline expression of GLUT1 or IRG1. Moreover, no changes were observed in SET7D or SDHB expression, indicating that oxidative phosphorylation pathways remained unchanged. Following LPS stimulation, monocytes from the nRVVac were inhibited from expressing IRG1. None of the other genes evaluated in this study were affected by this inflammatory challenge.

**Table 1:** Expression of genes related to cellular immunometabolism and epigenetic reprogramming in monocytes.

	Group	− LPS	+ LPS
**GLUT1**	**CTRL**	1.11 ± 0.22	1.29 ± 0.34
	**InVac**	2.93 ± 0.69	2.53 ± 0.67
	**nRVVac**	6.29 ± 3.0	3.01 ± 0.99
**HK2**	**CTRL**	1.15 ± 0.22	1.27 ± 0.34
	**InVac**	3.73 ± 0.92	1.85 ± 0.39
	**nRVVac**	5.36 ± 1.93 *	2.54 ± 1.25
**LDHA**	**CTRL**	1.02 ± 0.08	1.15 ± 0.24
	**InVac**	2.36 ± 0.53	1.71 ± 0.30
	**nRVVac**	4.75 ± 1.3 **	2.6 ± 0.96
**SET7D**	**CTRL**	1.03 ± 0.10	1.02 ± 0.09
	**InVac**	0.98 ± 0.13	0.99 ± 0.12
	**nRVVac**	0.82 ± 0.08	2.43 ± 1.89
**SDHB**	**CTRL**	1.02 ± 0.08	1.02 ± 0.07
	**InVac**	0.79 ± 0.04	0.86 ± 0.12
	**nRVVac**	0.94 ± 0.16	0.64 ± 0.18
**IRG1**	**CTRL**	1.80 ± 0.76	1.38 ± 0.38
	**InVac**	0.88 ± 0.41	0.74 ± 0.23
	**nRVVac**	0.23 ± 0.05	0.27 ± 0.09 *

Monocytes were isolated from PBMC of unvaccinated Controls (CTRL), vaccinated with inactivated vaccine (InVac), and non-replicating viral vector vaccine (nRVVac). Cells were challenged in vitro with LPS and collected for analyzing gene expression using qPCR. The baseline expression (− LPS) and gene expression after LPS stimulation (+ LPS) of GLUT1, HK2, LDHA, SET7D, SDHB, and IRG1 was calculated using the delta–delta CT method, comparing CT mean of CTRL group with CT of vaccinated groups. Data were expressed as means ± SEM of eight, seven, and six individual CTRL, InVac, and nRVVac donors. The results were analyzed using one-way ANOVA with Dunnett’s post-test. *p ≤ 0.05, **p ≤ 0.01.

### Vaccination induced histone modifications in *IL6*, *TNF*, *IL10*, and *CCL2* genes

**Figure 7:**
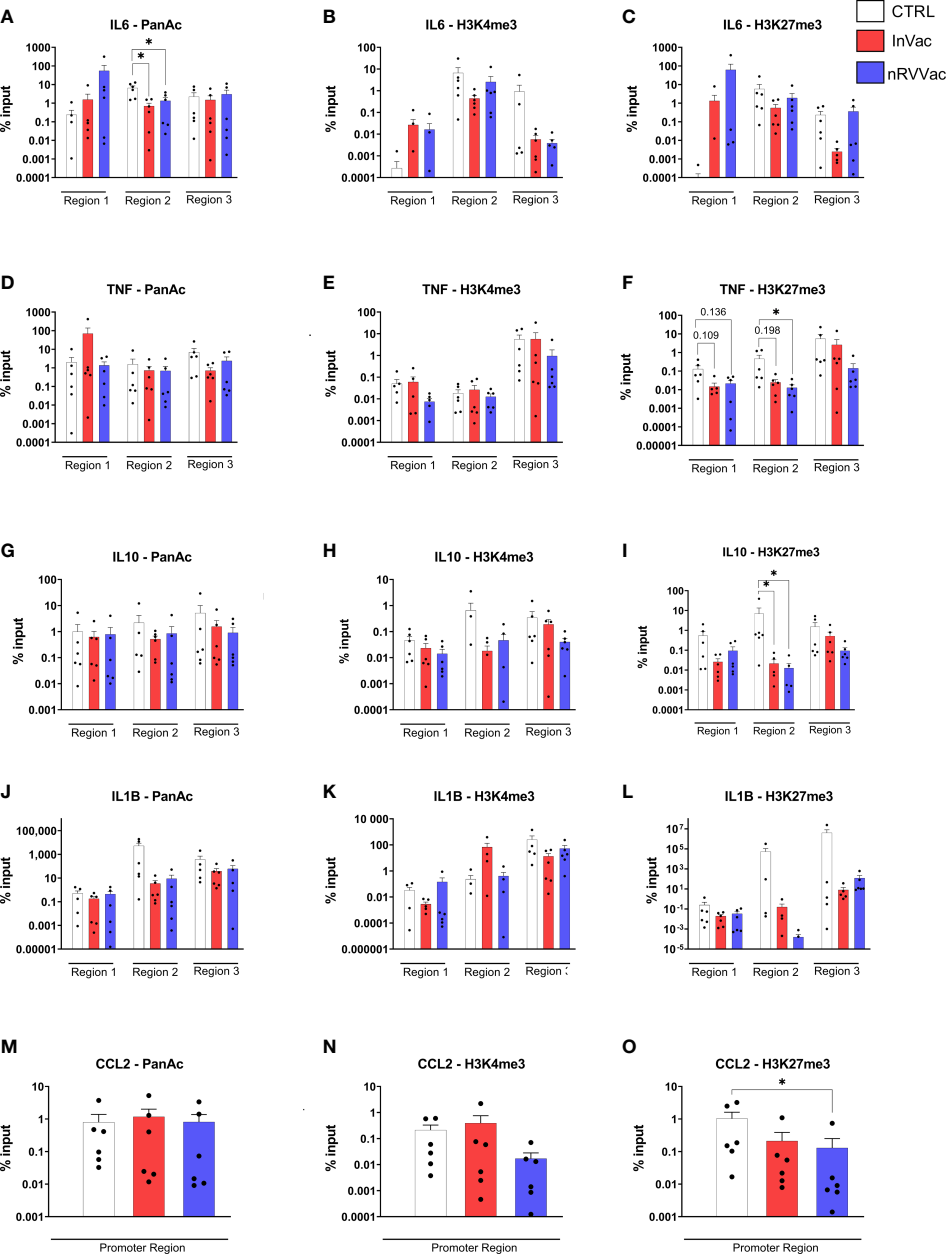
Histone modifications in frequently modified promoter regions of genes related to immune response. Monocyte chromatin was immuno-precipitated, and the DNA used for qPCR for *IL6* **(A–C)**, *TNF* **(D–F)**, *IL10* **(G–I)**, *IL1B* **(J–L)**, and *CCL2* **(M–O)** gene regions. The graphs represent the percentage of input of pan acetylation, H3K4me3, and H3K27me3 in each gene region, regarding non-precipitated samples. Individuals whose input values are 0 are not represented in the graph due to the logarithmic scale of the Y-axis but were included for statistical calculation. The results were analyzed by genic region using one-way ANOVA with multiple comparisons between all groups, followed by Tukey’s post-test, when the data was normally distributed, and Kruskal–Wallis test with multiple comparisons between all groups, followed by Dunn’s post-test, when the data was not normally distributed. Data were expressed as means ± SEM, n=6, *p ≤ 0.05.

While adaptive immune memory is marked by gene recombination, innate memory is related to the epigenetic modulation of gene transcription. Here, both COVID-19 vaccines reduced pan-acetylation of the *IL6* gene, suggesting that this genic region is less accessible in monocytes from vaccinated individuals (**Figure 7A**). Moreover, we evaluated two additional methylation sites, trimethylation of lysine 4 of histone H3 (H3K4me3) and trimethylation of lysine 27 of histone H3 (H3K27me3), which are associated with gene activation and repression, respectively. No changes were observed in H3K4me3 or H3K27me3 levels in IL-6 cells (**Figures 7B, C**).

Furthermore, a reduction in H3K27me3 levels was observed in monocytes after nRVVac vaccination, indicating that this gene was more accessible (**Figure 7F**). Interestingly, when analyzing the release of TNF-α by ELISA, we observed a reduction in this cytokine in the vaccinated subjects. However, no changes were observed in pan acetylation and H3K4me3 of *TNF* gene (**Figures 7D, E**). Considering the reduction in TNF-α release and the increased chromatin accessibility of this gene in monocytes from nRVVac subjects, we investigated the amount of mRNA in the cytosol to understand whether the monocytes of vaccinated subjects were transcribing cytokines. Therefore, we performed qPCR for the *TNF* genes and also for *IL6* and observed that, at basal levels, the mRNA production of both cytokines was unchanged (data not shown), but after stimulation with LPS, TNF-α mRNA levels were greater in vaccinated individuals, primarily in the InVac group (data not shown). This suggests that, although chromatin is more accessible for *TNF* transcription, the cytokine levels remained unchanged, probably because of a post-transcriptional control mechanism.

Another important gene involved in immune training immunity is *IL10*. H3K27me3 was reduced in the monocytes of both InVac and nRVVac groups, suggesting that this gene is more accessible in vaccinated subjects (**Figure 7I**). Furthermore, pan-acetylation or H3K4me3 levels remained unchanged (**Figures 7G, H**). Moreover, we analyzed histone modifications in the promoter region of *CCL2* (**Figures 7M–O**) and observed a reduction in H3K27me3 in the monocytes of nRVVac subjects, suggesting that this region is more accessible for transcription (**Figure 7O**). Finally, pan acetylation or H3K4me3 levels in *CCL2* were unchanged. We also analyzed histone modifications in *IL1B*, but no changes were observed (**Figures 7J–L**).

## Discussion

This study was conducted with aim of analyzing whether COVID-19 vaccines have the ability to induce trained immunity, as has been observed for other vaccines for human diseases. We assessed the results of phenotype characterization of monocytes isolated from vaccinated subjects who were subjected to full immunization protocols with an inactivated vaccine or a non-replicating viral vector vaccine. Both groups were defined based on currently available vaccines in Brazil, where the study was conducted. COVID-19 has been characterized as a disease that is markedly inflammatory, with a cytokine storm that is deleterious for patients with severe clinical presentation (Fara et al., 2020; Kim et al., 2020; Manjili et al., 2020; Wang and Perlman, 2022).

The vaccine became globally available at the end of 2020 and in Brazil from January 2021, when the population could receive an inactivated vaccine (CoronaVac—Sinovac/Butantan) and a non-replicating viral vector vaccine (ChAdOx1 nCoV-19—Oxford/AstraZeneca/FioCruz). Recently, other RNA vaccines have become available; however, in this study, our cohort was comprised of the first two vaccines. Vaccination was advantageous primarily to control the development of severe forms of disease and death, which was true for both inactivated and viral vector vaccines (Bayhan and Guner, 2022; Cerqueira-Silva et al., 2022; Sevinc et al., 2022; Promlek et al., 2023; Shi et al., 2023; Wei et al., 2023). The adaptive immune response induced by these vaccines, production of neutralizing antibodies, and cellular response have been extensively studied (Escobar et al., 2022; Kim et al., 2022; Sheng et al., 2022; Wang et al., 2022; Zhao et al., 2022). Furthermore, the understanding of COVID-19 pathology is evolving since its emergence in 2019 (Mazza et al., 2022; Shafqat et al., 2022; Davis et al., 2023). Here, we sought to understand whether vaccination could interfere with innate immunity and consequently regulate the exacerbated cytokine storm caused by the virus. Then, our initial objective was to analyze whether the cells from immunized donors were already trained due to the vaccination or not.

*In vitro* training of human monocytes is well stablished and consists in a first stimulus with β-glucan, the Bacillus Calmette–Guérin (BCG) vaccine, or oxidized low-density lipoprotein (oxLDL). *In vivo*, vaccines could serve as a first stimulus. After the first stimuli, a challenge with LPS is used as an unrelated secondary bacterial stimulus (Bekkering et al., 2016). To evaluate the extension of training, we analyzed if monocytes from unvaccinated and vaccinated donors presented altered phenotype under the challenge with LPS. Moreover, the training phenotype is not limited to just one feature but actually to a set of markers that we showed throughout this study.

We observed an increase in the frequency of classical monocytes in InVac-immunized individuals following stimulation with LPS. The same result was observed in the literature using another inactivated vaccine against COVID-19 (Liu et al., 2021). Classical monocytes are the main cells involved in phagocytosis, production of reactive oxygen species, high production of IL-10, and low production of TNF-α in response to LPS. The increase in this population in the InVac group could favor the control of the inflammatory response (Yang et al., 2014). Moreover, CD163 is a scavenger receptor involved in regulating the immune response and resolution of inflammation (Onofre et al., 2009) and is also a marker that characterizes trained cells (Quintin et al., 2012; Guha et al., 2021). We observed an increase in classical and intermediate monocytes from the nRVVac-vaccinated group, suggesting that monocytes vaccinated with nRVVac are trained for the regulatory profile. It is important to note that LPS challenge activates and stimulates classical monocyte survival and causes a reduction in CD163 expression (Onofre et al., 2009). Therefore, we observed that the frequency of CD163 expression in classic monocyte is increased, accompanying the increase in the frequency of this population; however, the MFI of this marker showed a reduction after stimulation with LPS. In addition, after the stimulus with LPS, there was a reduction in the frequency of intermediate monocytes; consequently, there was a reduction in the frequency of CD163 and in the MFI due to the stimulus with LPS in CTRL group, indicating that both vaccines are playing a role in monocytes profile differentiation.

To concatenate all the immunophenotyping data and provide an overall view of our findings, we used tSNE as an integrative data analysis tool. For this, we considered CD163 as a marker that was increased in trained cells and therefore considered the starting point; monocytes from vaccinated individuals, after stimulation with LPS, presented a down-modulation of clusters that did not express CD163. More clusters, specifically Pop1 and Pop2, expressed this marker. In addition, cells that express more CD163 also expressed the highest levels of CD16, CD86, and HLA-DR, demonstrating that they are monocytes with an antigen-presenting function, a typical function of intermediate monocytes (Jakubzick et al., 2017), which could be advantageous post-immunization. We also observed that before stimulation with LPS, all individuals had the same MFI and frequency of expression of surface markers, except for CD163 in classic monocytes, suggesting that the basal profile of monocytes from different experimental groups was similar, but they responded differently when stimulated, suggesting that monocytes from vaccinated individuals were less inflammatory than those from non-immunized individuals, showing a regulatory phenotype. In the context of COVID-19, the balance of cytokines determines the course of the disease (Xu et al., 2020). Vaccines could help regulate the exacerbated production of cytokines by monocytes, thus avoiding high levels of inflammation when the vaccinated individual encounters SARS-CoV-2.

Another evidence that reinforces the regulatory phenotype found in monocytes from vaccinated individuals is the reduction in IL-6 production in the InVac group and reduction in TNF-α in both the vaccinated groups. Although the nRVVac group did not show a reduction in IL-6, it was the only group that showed an increased expression of IL-10, an important regulatory cytokine (Ip et al., 2017). Indeed, IL-6 and TNF-α are pro-inflammatory cytokines involved in the immunopathology of COVID-19; IL-6, in particular, can lead to macrophage activation syndrome and cytokine storm, the most serious complications of COVID-19 (Qin et al., 2020; Tan et al., 2020). Between other inflammatory cytokines, IL-6 and TNF-α are downregulated and IL-10 can be upregulated in monocytes trained for the regulatory profile (Cauchi and Locht, 2018; Quinn et al., 2019; Guha et al., 2021).

Moreover, all groups produced the same levels of IL-1β, and only the InVac group did not increase IL-1Ra production after stimulation with LPS. Therefore, our results differ from others in the literature that demonstrated that cells trained with a regulatory profile release less IL-1β when trained by natural infection with *Plasmodium falciparum* (Guha et al., 2021) and more IL-1Ra when trained with helminth extract (Quinn et al., 2019). Considering that we were comparing with a vaccine and that the supposed training took place *in vivo*, the type of stimulus likely influenced which genes are affected during training-induced chromatin remodeling, which will determine how the cell will respond when challenged.

In addition to cytokine production, we also evaluated the gene expression of proteins related to immunometabolism and the tolerance phenotype, such as IRG1 (Li et al., 2013), to confirm whether the reduction in IL-6 and TNF-α production was not associated with the tolerance phenotype (Li et al., 2013). Also associated with epigenetic changes and contrary to training, cells of the innate immune system can be suppressed after prolonged exposure to an infectious agent, persistent pro-inflammatory signals, or specific antigens such as LPS. In monocytes, this phenomenon is called immune tolerance (Li et al., 2013). Although in the trained regulator profile, there is also a reduction in the production of pro-inflammatory cytokines, it differs from the tolerance phenotype, as tolerant cells increase the expression of immunoresponsive gene 1 (IRG1) (Li et al., 2013), which causes immunometabolic changes associated with itaconate pathway that leads the monocyte to the state of immunoparalysis (Domínguez-Andrés et al., 2019). This phenotype is characterized by reduced production of pro-inflammatory cytokines but without increasing the production of regulatory cytokines and the expression of membrane proteins that characterize the trained cell (Li et al., 2013). We observed a reduction in IRG1 expression in the nRVVac group and maintenance of expression levels in the InVac group, demonstrating that the reduction in pro-inflammatory cytokine production was not caused by tolerization. Additionally, an increase in the aerobic glycolysis pathway was associated with the training phenotype (Cheng et al., 2014; van der Meer et al., 2015). Trained monocytes from individuals vaccinated with BCG showed increased expression of GLUT1, LDHA, and HK2 (Kühtreiber et al., 2020). Here, we observed an increased expression of glycolysis-related proteins in the nRVVac group. Moreover, we did not observe alterations in the expression of SET7 and SDHB proteins, which are related to the induction of the pro-inflammatory training phenotype (Keating et al., 2020), reinforcing the idea that monocytes from vaccinated groups may be trained to the regulatory phenotype. Altogether, the immunophenotype, cytokine production, and immunometabolic control could indicate the polarization of the training phenotype to a regulatory milieu.

Chromatin remodeling of cytokine gene promoters or transcription factors is a hallmark of innate training that suppresses the antigen receptor somatic recombination seen in the adaptive immune response (Van Der Heijden et al., 2018; Kar and Joosten, 2020). Hence, we performed a chromatin immunoprecipitation assay followed by specific gene amplification to evaluate which gene promoters or regions of promoters were under epigenetic control in monocytes from vaccinated subjects. Here, we evaluated three parameters of epigenetic modulation: pan acetylation and H3K4me, which are often associated with gene activation and increased DNA accessibility (Van Der Heijden et al., 2018), and H3K27me3, which is associated with gene repression and DNA inaccessibility (Van Der Heijden et al., 2018). A reduction in pan acetylation was observed for the *IL6* gene in both vaccinated groups, with unchanged H3K4me3 levels. These data are consistent with the reduction in IL-6 release observed in vaccinated individuals. Guha et al. (2021) trained monocytes for the regulatory profile using *Plasmodium falciparum*-infected red blood cells as a stimulus and observed an increase in chromatin accessibility for IL-6 transcription through an increase in H3K4me3. However, 5 days after challenge with *P. falciparum*-infected red blood cells, they observed a reduction in H3K4me3 and, consequently, a decrease in chromatin accessibility in the *IL6* gene (Guha et al., 2021). In our results, despite the absence of alterations in H3K4me3 of *IL6*, we observed chromatin closure due to a reduction in pan acetylation in vaccinated individuals, corroborating the regulatory phenotype.

Interestingly, we observed that although TNF-α was suppressed in vaccinated individuals, the *TNF* gene was more accessible for transcription in nRVVac-vaccinated individuals. Using qPCR of the *TNF* genes and *IL6*, we evaluated if the genes were transcribed more or less and verified that the transcription of *IL6* did not differ between the groups, both basal and stimulated. However, when we analyzed the *TNF* gene, we detected an increase in the transcription of this cytokine when stimulated with LPS. These data suggest that although TNF-α transcription is increased, its translation or release may be regulated by a post-transcriptional mechanism.

Regarding *IL1B*, the promoter regions evaluated here were not under epigenetic control, which is in line with IL-1β production by monocytes, although other studies using trained cells in the regulatory profile showed a reduction in IL-1β release (Quinn et al., 2019; Murphy et al., 2023). The *IL10* gene showed an increase in chromatin accessibility in both vaccinated groups with reduced H3K27me3, although only the nRVVac group showed an increase in *IL10* cytokine release, probably due to its delayed release kinetics, which increased in a time-dependent manner, with a release peak between 20 and 48 h after stimulation with LPS (Malefyt et al., 1991; Amoroso et al., 2012). Finally, we observed enhanced chromatin accessibility in the *CCL2* gene, mediated by a reduction in H3K27me3. The increased release of this chemokine has been reported in monocytes from individuals trained with the adenoviral vector COVID-19 vaccine to a regulatory profile (Murphy et al., 2023) and in trained monocytes with lipoprotein to a pro-inflammatory phenotype (Groh et al., 2018). The increased production of this chemokine enhances the LPS-induced production of IL-10 and causes increased migration of immune cells to a site with an infectious agent, which may favor a rapid response and resolution of the infection (Sierra-Filardi et al., 2014; Gschwandtner et al., 2019).

In our cohort, we compared two types of vaccines and demonstrated that inactivated COVID-19 vaccines trained human monocytes to a regulatory phenotype, mediated by histone modifications in the *IL6* and *IL10* genes, whereas the non-replicating viral vector COVID-19 vaccine drove human monocytes to a regulatory phenotype, mediated by histone modifications in the *IL6*, *IL10*, *TNF*, and *CCL2* genes. Our data collectively suggest that both vaccines may be associated with the training of monocytes, leading them to adopt a regulatory phenotype. We demonstrated that each vaccine activates distinct chromatin remodeling regions, resulting in different immune responses and metabolic regulation. These differences could potentially contribute to the effectiveness of vaccines in controlling excessive inflammation upon subsequent SARS-CoV-2 infection in patients.

## Materials and methods

### Ethical aspects and donors

This study was approved by the Research Ethics Committee (CEP) of the School of Pharmaceutical Sciences of Ribeirão Preto (CAAE 30736120.7.0000.5403). All individuals who agreed to participate in the study signed a Free and Informed Consent Form that was filed in the laboratory. Individuals were recruited between 01/02/2021 and 01/05/2021 when they donated platelets via apheresis at the Blood Bank of Ribeirão Preto. After the declaration of interest in participating in the research and signing the TCLE, the leukocyte reduction chamber was collected where the donor’s leukocytes were present.

For the group of unvaccinated control individuals, filters were collected only from healthy donors, between 18 and 60 years old, of both sexes and belonging to any ethnicity, prior to COVID-19 vaccination. Individuals who had infections or chronic diseases, people living with HIV, pregnant women, individuals with flu-like symptoms or diagnosed COVID-19 <150 days ago, people who were vaccinated against COVID-19 <150 days ago, and those who did not consent to participate in the study were not included in this study.

For the group of vaccinated individuals, filters were collected only from healthy donors who completed the two-dose vaccination schedule of the same vaccine (inactivated vaccine CoronaVac, Sinovac/Butantan or non-replicating viral vector vaccine ChAdOx1 nCoV-19, Oxford/AstraZeneca/FioCruz) at least 15 days and up to 150 days ago, aged between 18 and 60 years, of both sexes and belonging to any ethnicity. Individuals who had infections or chronic diseases, people living with HIV, pregnant women, individuals with flu-like symptoms or diagnosed with COVID-19 <150 days ago, and those who did not consent to participate in the study were not included. There is still no evidence demonstrating that natural infection by SARS-CoV-2 causes training, but we established a cutoff time of 150 days (5 months) for individuals who had flu-like symptoms or who were vaccinated for COVID-19 (for CTRL subjects), as the work that shows persistence of immunity for the longest time was 90 days after training (Cirovic et al., 2020).

### PBMC collection and isolation of monocytes

Cells contained inside the filter were collected by washing with phosphate-buffered saline solution (PBS) and gently transferred to Ficoll-Paque Plus™, d = 1.078 g/ml (GE Healthcare Bio-Science AB, Uppsala, Sweden) and centrifuged at 600×*g* for 30 min at 18°C. Peripheral blood mononuclear cells (PBMCs) were collected and washed twice with PBS and once with red blood cells lysis buffer. PBMCs were cryopreserved at a concentration of 1.5×10^8^ PBMC/ml in liquid nitrogen, where they were stored until use. For monocyte isolation, after thawing, 3.5×10^6^ monocytes were obtained through magnetic immunoselection using CD14+ beads (CD14 MicroBeads Human Miltenyi Biotec) and an LS magnetic column (Miltenyi), according to the manufacturer’s instructions.

### *In vitro* stimulation of monocytes

Isolated monocytes (5×10^5^) were plated per well in 96-well plates containing AIM-V medium (AIM-V ^®^ Medium Gibco). The cells were incubated at 37°C and 5% CO_2_ for 1 h to allow monocytes to adhere to the plate. Then, the culture medium was replaced with fresh AIM-V for the non-stimulated group and AIM-V + 100 ng/ml LPS for the stimulated group as previously described (Garcia-Valtanen et al., 2017; Walachowski et al., 2017; Quinn et al., 2019; Peng et al., 2022), and incubated for 6 h.

### Flow cytometry and tSNE analysis

After 6 h of cultivation, the cells were collected, stained with Fixable Viability Stain 780 (BD Biosciences). Monocytes suspended in PBS were incubated with combined antibodies as follows: CD14 (PerCP-Cy5.5, clone M5E2, BD Biosciences, Cat. no. 550787), CD16 (PE-Cy7, clone 3G8; BD Biosciences, Cat. no. 557744), CD45 (APC-Cy7, clone 30-F11; BD Biosciences, Cat. no. 557659), CD86 (PE, clone IT2.2; BD Biosciences, Cat. no. 555665), CD163 (Alexa Fluor 647, clone GHI/61, BD Biosciences, Cat. no. 562669), HLA-DR (V450, clone L243, BD Biosciences, Cat no. 642276), and CD206 (Alexa Fluor 488, clone 15-2, Biolegend, Cat. no. 321114). Antibodies were used according to the manufacturer’s instructions. From each sample, 10,000–100,000 events were acquired using the LRS II Fortessa cytometer (BD Biosciences, San Diego, CA, USA), and the analyses were performed using BD FACSDiva 8.0.1 and FlowJo software version 10.8.1. All the gate strategies are described in **Supplementary Material S3**. For tSNE analysis, FlowJo software version 10.8.1 was used. From the gate of viable monocytes, 5,000 events were automatically extracted from each sample using the plug-in DownSample v.3.3.1, generating a representative population of the sample. All tSNE parameters are described in **Supplementary Material S3**.

### ELISA

Quantification of cytokines IL-1β, TNF-α, IL-6, IL-10, and IL-1Ra (R&D Systems, Mineapolis, EUA) release was performed using enzyme-linked immunosorbent assay, according to the manufacturer’s instructions.

### RNA extraction and qPCR

RNA was extracted from monocytes using the TRIzol reagent (Thermo Fisher) according to the manufacturer’s instructions. RNA content and quality were quantified and assayed using a Nanodrop (Thermo Fisher Scientific), and RNA was reverse-transcribed using a High-Capacity cDNA Reverse Transcription Kit (Applied Biosystems). qPCR for SYBR green primers (LDHA, GLUT1, HK2, SDHB, SET7D, IRG1, and ACTB) was performed using 2× qPCRBIO Sygreen Mix Hi-ROX (PCR Biosystem) on a StepOne Plus Real-Time PCR System (Applied Biosystems), and qPCR for TaqMan primers (ACTB, IL6, and TNF) was performed using 2× GoTaq Probe qPCR Master Mix (Promega) on a StepOne Plus Real Time PCR System (Applied Biosystems). The human probes for the TaqMan primers were [HEX]ACCACGCTCTTCTGCCTGCTGCACT[OQA] for TNF, [HEX]TGCTCCTGGTGTTGCCTGCTGCCTT[OQA] for IL6, and [6FAM]CCAGCCATGTACGTTGCTATCCAGGC[TAM] for ACTB. The cycle threshold (Ct) values were analyzed by the comparative Ct (ΔΔCt) method and normalized to the endogenous control β-actin (ACTB). Fold difference was calculated as 2^−ΔΔCt^. Nucleotide sequences of the primers used are listed in the supplemental material.

### Chromatin imunoprecipitation

To assess the epigenetic markers associated with inflammatory changes in monocytes before challenge with LPS, 1×10^6^ of isolated monocytes was fixed in 18.5% formaldehyde for 10 min and quenched with glycine 10× for 5 min. Chromatin was sonicated from these cells using a Bioruptor Pico^®^ (Diagenode) for 11 cycles of 15 s ON, 90 s OFF, at 4°C. Chromatin immunoprecipitation (ChIP) was performed using the following antibodies: IgG control (Rabbit Control IgG ChIP Grade, Abcam), H3K4me3 (Abcam), H3K27me3 (Abcam), and H3K9+K14+K18+K23+K27Ac (Abcam).

ChIP-treated DNA was processed for qPCR analysis using SYBR green reagents, as was described previously, for 50 cycles. Primers used for this reaction are listed in the supplemental material. Finally, for all ChIP experiments, qPCR values were normalized to the percent recovery of the input DNA.

### Data analysis

All results were statistically analyzed using GraphPad Prism software version 9.0, with p-values ≤0.05 considered significant (Graph Pad Software Inc., San Diego, USA). Data are shown as means ± SEM.

For flow cytometry analysis, we used two-way analysis of variance (ANOVA) with multiple comparisons between all groups, followed by Tukey’s *post-hoc* test. For ELISA, we used one-way ANOVA for multiple comparisons between all groups, followed by Šidák’s *post-hoc* test. For qPCR analyses, a one-way ANOVA was used to compare the InVac and nRVVac groups with the CTRL group, followed by Dunnett’s *post-hoc* test.

For the ChIP results, when the data were normally distributed, we used a one-way ANOVA with multiple comparisons between all groups, followed by Tukey’s *post-hoc* test. When the data were not normally distributed, we used the Kruskal–Wallis test with multiple comparisons between all groups, followed by Dunn’s post-test.

## Data availability statement

The original contributions presented in the study are included in the article/**Supplementary Material**. Further inquiries can be directed to the corresponding author.

## Ethics statement

This study was approved by the Research Ethics Committee (CEP) of the School of Pharmaceutical Sciences of Ribeirão Preto (CAAE 30736120.7.0000.5403). The patients/participants provided their written informed consent to participate in this study.

## Author contributions

MA: conceptualization, methodology, validation, formal analysis, investigation, data curation, writing—original draft, writing—review and editing, and visualization. FS, HG and RC: conceptualization, methodology, validation, formal analysis, investigation, and writing—review and editing. FM, JL and CF: methodology, validation, and writing—review and editing. FF: conceptualization, methodology, resources, writing—review and editing, supervision, project administration, and funding acquisition. All authors contributed to the article and approved the submitted version.
