# Supplementary material for: COVID-19 inactivated and non-replicating viral vector vaccines induce regulatory training phenotype in human monocytes under epigenetic control
Source: Front Cell Infect Microbiol. 2023 Jul 14;13:1200789. doi: 10.3389/fcimb.2023.1200789 (PMC10382685; doi:10.3389/fcimb.2023.1200789)
Supplement: Supplementary file 1 [file DataSheet_1.pdf]

Supplementary Material

COVID-19 inactivated and non-replicating viral vector vaccines induce regulatory training phenotype in human monocytes under epigenetic control

Mateus da Silva Matias Antunes, Fabrícia Heloisa Cavicchioli Sugiyama, Humberto Doriguetto Gravina, Ricardo Cardoso Castro, Francisco Javier Romero Mercado, Julia Oliveira de Lima, Caroline Fontanari, Fabiani Gai Frantz*.

*** Correspondence:** Fabiani Gai Frantz (E-mail: frantz@usp.br)

Supplementary Material 1

Characterization of the unvaccinated control group, based on gender, age, occurrence of COVID-19, occurrence of flu-like symptoms in the last 6 months and time elapsed after vaccination for COVID-19.

CODE	SEX	AGE	HAD COVID-19? WHEN?	FLU SYMPTOMS IN THE LAST 6 MONTHS?	ESTIMATED TIME VACCINATED FOR COVID-19? (2° DOSE)
CTRL 1	Male	31	Yes (270 days)	SIM, (60 days, but tested negative for COVID-19)	NA
CTRL 2	Male	33	Yes (210 days)	No	NA
CTRL 3	Male	30	Yes (450 days)	No	NA
CTRL 4	Male	37	No	No	NA
CTRL 5	Female	32	No	No	NA
CTRL 6	Male	44	Yes (180 days)	No	NA
CTRL 7	Male	23	No	No	NA
CTRL 8	Male	27	No	No	NA
CTRL 9	Male	27	No	No	NA
CTRL 10	Male	30	No	No	NA
CTRL 11	Male	22	No	No	NA
CTRL 12	Male	29	No	No	NA
CTRL 13	Male	29	No	No	300 days (inactivated vaccine)
CTRL 14	Male	23	No	Yes (150 days, but tested negative for COVID-19)	NA
**Mean**		**29.7**			

NA = Not applicable, i.e. not vaccinated.

Supplementary Material 2

Characterization of the vaccinated individuals, based on gender, age, occurrence of COVID-19, occurrence of flu symptoms in the last 6 months and time elapsed after vaccination for COVID-19.

CODE	SEX	AGE	HAD COVID-19? WHEN?	FLU SYMPTOMS IN THE LAST 6 MONTHS?	ESTIMATED TIME VACCINATED FOR COVID-19? (2° DOSE)
InVac 1	Male	24	No	No	120 days
InVac 2	Male	48	No	No	150 days
InVac 3	Male	39	Yes (480 days)	No	150 days
InVac 4	Male	29	No	No	120 days
InVac 5	Male	50	No	No	30 days
InVac 6	Male	46	No	No	30 days
InVac 7	Male	34	No	No	15 days
InVac 8	Male	33	*MISSING*	*MISSING*	60 days
InVac 9	Male	34	No	No	30 days
InVac 10	Male	20	No	No	30 days
**Mean**		**35.7**			**70.5 days**

Missing = information loss.

	CODE	SEX	AGE	HAD COVID-19? WHEN?	FLU SYMPTOMS IN THE LAST 6 MONTHS?	ESTIMATED TIME VACCINATED FOR COVID-19? (2° DOSE)
	nRVVac 1	Male	45	No	No	60 days
	nRVVac 2	Male	45	Yes (240 days)	No	30 days
	nRVVac 3	Male	56	No	No	20 days
	nRVVac 4	Male	38	No	No	90 days
	nRVVac 6	Male	24	Yes (420 days)	No	120 days
	nRVVac 7	Male	38	No	No	60 days
	nRVVac 8	Male	50	No	No	150 days
**Mean**	**nRVVacs**		**42.2**			**66.25 days**

Supplementary Material 3. Genes analyzed by qPCR and their respective primers and transcription products, in base pairs (bp), for gene expression analysis.

Gene	Foward (5'-3')	Reverse (3'-5')	Product
	**SYBRGREEN PRIMERS**		
LDHA	ATCTTGACCTACGTGGCTTGGA	CCATACAGGCACACTGGAATCTC	180 pb
GLUT1	ACTCCATCATGGGCAACAAG	TGCCGACTCTCTTCCTTCAT	220 pb
HK2	CCACCTTTGTGAGGTCCACT	GGAGCCCATTGTCCGTTACT	114 pb
SDHB	ACAGCTCCCCGTATCAAGAAA	GCATGATCTTCGGAAGGTCAA	177 pb
SET7D	AGTGTAAACTCCCTGGCCCT	GTTCACGGAGAAAAGAACGG	105 pb
IRG1	TGAACGGTGTGGCTATTCACT	ACTTTGGACTCCTTGGCAGG	123 pb
ACTB	CCAGCCTTCCTTCCTGGGCAT	AGGAGCAATGATCTTGATCTTCATT	211 pb
	**TAQMAN PRIMERS**		
ACTB	GCGAGAAGATGACCCAGATC	CCAGTGGTACGGCCAGAGG	103 pb
IL6	AGAAGCTCTATCTCCCCTCC	TTTGGAATCTTCTCCTGGGG	305 pb
TNF	CTTCTCCTTCCTGATCGTGG	ATTAGAGAGAGGTCCCTGGG	711 pb

Supplementary Material 4. Genes analyzed by qPCR and their respective primers and transcription products, in base pairs (bp), for ChIP analysis.

Gene	Foward (5'-3')	Reverse (3'-5')	Product
IL1B Primer 1	TCTGAATAAGAGGAAAGTGGTAAC	AAGAAAGTTGAGCAGGAGAGAA	140 pb
IL1B Primer 2	GCAAATGGGGAGAAAAGACT	TCCACTGACCTGTAATAAGCC	255 pb
IL1B Primer 3	GGACTTCTCTTTCACACATTCAT	ATTCTCTTCAGCCAATCTTCAT	165 pb
IL6 Primer 1	TGTGAGCGGCTGTTGTAGAA	TGCGATGGAGTCAGAGGAAA	115 pb
IL6 Primer 2	TTGGGGGTTGAGACTCTAATA	GGAACTGCCAGCGGCGGTCG	179 pb
IL6	GGCGGCTACATCTTTGGAAT	CTGAGAAAGGAGGTGGGTAGG	116 pb

Primer 3			
TNFA Primer 1	GGGGGTCTGTAGTTGCTTCT	TGTTCCTCAGCCTCTTCTCC	240 pb
TNFA Primer 2	GTGGGAGAGTGGATGAAGGC	GATAGGGAGGGATGGAGAGA	100 pb
TNFA Primer 3	TCTCTCCTTATCTCCCCCATCT	AACCAGACACCTCAGGGCTAA	133 pb
IL10 Primer 1	CCTACAGACCAGCAGGGACA	CAGGAGCCAAAGGTGAGTGA	250 pb
IL10 Primer 2	AGCAGCCAGAGGGTTTACAA	CAGGAAGAACAAAAGGAGAATG	244 pb
IL10 Primer 3	GTGAGTATGATTCCTTCCTGTCC	GCAAGCCCCTGATGTGTAGA	250 pb
CCL2 PRIMER	CCCATTTGCTCATTTGGTCTCAGC	GCTGCTGTCTCTGCCTCTTATTGA	210 pb
